# Novel All Trans-Retinoic Acid Derivatives: Cytotoxicity, Inhibition of Cell Cycle Progression and Induction of Apoptosis in Human Cancer Cell Lines

**DOI:** 10.3390/molecules20058181

**Published:** 2015-05-07

**Authors:** Ebtesam Saad Al-Sheddi, Mai Mohammad Al-Oqail, Quaiser Saquib, Maqsood Ahmed Siddiqui, Javed Musarrat, Abdulaziz Ali Al-Khedhairy, Nida Nayyar Farshori

**Affiliations:** 1 Department of Pharmacognosy, College of Pharmacy, King Saud University, Riyadh 11495, Saudi Arabia; E-Mails: ebtesam.saad@yahoo.com (E.S.A.-S.); maioqail@hotmail.com (M.M.A.-O.); 2 Zoology Department, College of Science, King Saud University, Riyadh 11451, Saudi Arabia; E-Mails: quaiser.saquib0@gmail.com (Q.S.); maqsoodahmads@gmail.com (M.A.S.); musarratj1@yahoo.co.in (J.M.); kedhairy@yahoo.com (A.A.A.-K.); 3 Al-Jeraisy Chair for DNA Research, College of Science, King Saud University, Riyadh 11451, Saudi Arabia

**Keywords:** all trans-retinoic acid, anticancer activity, apoptosis, cell cycle arrest, cytotoxicity

## Abstract

Owing to the pharmacological potential of ATRA (all trans-retinoic acid), a series of retinamides and a 1-(retinoyl)-1,3-dicyclohexylurea compound were prepared by reacting ATRA with long chain alkyl or alkenyl fatty amines by using a 4-demethylaminopyridine (DMAP)-catalyzed *N,N*'-dicyclohexylcarbodiimide (DCC) coupling. The successful synthesis of the target compounds was demonstrated using a range of spectroscopic techniques. The cytotoxicity of the compounds was measured along with their ability to induce cell cycle arrest and apoptosis in human cancer cell lines MCF-7 (breast cancer) and HepG2 (liver cancer) and normal human cell line HEK293 (embryonic kidney). The results of cytotoxicity and flow cytometry data showed that the compounds had a moderate to strong effect against MCF-7 and HepG2 cells and were less toxic to HEK293 cells. *N*-oleyl-retinamide was found to be the most potent anticancer agent and was more effective against MCF-7 cells than HepG2 cells.

## 1. Introduction

Non-contagious diseases, such as cancer, are responsible for 64% of deaths worldwide [1]. Cancer involves an unrestrained, rapid increase in the number of diseased cells, which affects healthy cells adversely, a process that ultimately leads to the death of the cancer patient. Differentiation therapy, first proposed by Sachs [2], has been extensively studied as a means of treating cancer. All trans-retinoic acid (ATRA) is known to induce differentiation in various cancer cells [3,4,5,6]. The cytotoxic responses of ATRA against various cancerous cell lines have also been reported [7]. The physiological activity of retinoids is due to their interaction with two types of receptors: the retinoic acid receptors (RARα, RARβ and RARγ) and the retinoid X receptors (RXRα, RXRβ and RXRγ). Retinoids bind to RAR/RXR heterodimers at the retinoic acid response element (RARE), induce conformational changes and thereby lead to transcriptional activation of the target genes [8]. However, the clinical applications of ATRA have been limited by epigenetic changes that can eventually make cells resistant. Therefore, it is vital to improve the effectiveness of ATRA through structural modifications.

By chemically modifying the polar carboxylic acid group in ATRA, new retinoids with high potency and low toxicity can be obtained [9,10,11,12]. Furthermore, the attachment of fatty acids may lead to drugs with enhanced stability, solubility, cellular uptake and shorter half-lives. For example, linking paclitaxel to a natural fatty acid was shown to increase its accumulation in tumors [13], and the fatty acylamide derivative of doxorubicin was found to be more effective than the unmodified parent compound in inhibiting the proliferation of ovarian and colon cancer cells [14]. In addition, conjugating fatty acyl groups to cytarabine improved its cellular uptake [15].

In view of the above-mentioned examples, it was speculated that the effectiveness of ATRA could be improved by conjugating its polar carboxylic acid group to various long chain fatty acid amines. These novel hybrids were prepared and then tested against two human cancer cell lines—MCF-7 (breast cancer) and HepG2 (liver cancer)—to determine their cytotoxicity, their ability to inhibit cell cycle progression and their effect on apoptosis. In addition, the cytotoxic effect of the compounds against the normal cell line HEK293 (human embryonic kidney) was also evaluated.

## 2. Results and Discussion

Vitamin A, its derivatives and its active metabolite ATRA play a pivotal role in cell growth, differentiation, apoptosis and other related processes [16,17]. Although the retinoids are potential chemotherapeutic and chemopreventive agents [18,19], their usefulness is currently limited by their side effects [20,21,22,23,24]. Therefore, the synthesis of new retinoid hybrids is vital. This work describes the synthesis, characterization, cytotoxicity, effect on cell cycle progression and apoptotic effect of various derivatives of ATRA.

### 2.1. Chemistry

**Scheme 1:**
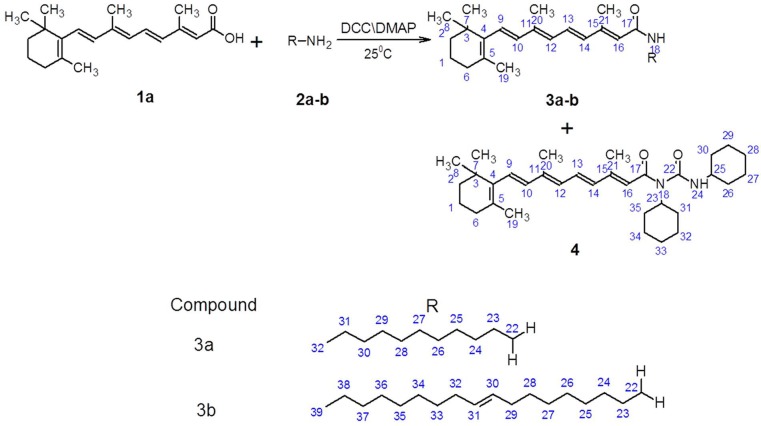
Reaction scheme showing the synthesis of novel all trans-retinoic acid (ATRA) derivatives. Compounds **3a**, **3b** and **4** were prepared by a 4-demethylaminopyridine (DMAP)-catalyzed *N,N*';-dicyclohexylcarbodiimide (DCC) coupling reaction in DMSO.

The chemical reaction sequence has been outlined in Scheme 1. ATRA reacts with long chain fatty amines in the presence of *N,N*'-dicyclohexylcarbodiimide (DCC) and 4-demethylaminopyridine (DMAP) in DMSO to yield the amidic derivatives **3a**, **3b** and the 1-substituted-1,3-dicyclohexylurea derivative **4** of all trans-retinoic acid (ATRA).

During DCC-catalyzed amide synthesis, the side product is dicyclohexylurea [25]. However, under suitable reaction conditions, a satisfactory yield of both an amide derivative and an acyl urea derivative can be achieved. The optimum conditions were determined using various ratios of reagent and catalyst. The generality and scope of the synthetic procedure was demonstrated using both a long chain alkylamine and a long chain alkenyl amine. The structure of compounds **3a**, **3b** and **4** was established using their spectral data.

The IR of compound **3b** revealed characteristic absorption bands at 3302 (N-H) and 1633 cm^−1^ (C=O). ^1^H-NMR was informative, and characteristic resonances at 7.25 (s, 1H, NH), 6.79 (dd, 1H, C*^13^*H), 6.23 (m, 2H, C*^9^*H, C*^12^*H), 6.09 (d, 1H, C*^10^*H), 5.74 (s, 1H, C*^16^*H), 5.65 (s, 1H, C*^14^*H) and 5.30 ppm (m, 2H, C*^30^*H = C*^31^*H) were observed. These were correlated with resonances in the ^13^C-NMR spectrum at 140.65, 137.20, 130.32, 130.04, 129.58, 125.74 and 126.10 ppm. Besides these, a few other significant carbon signals, such as a characteristic peak at δ_c_166.80 (C=O), were observed. Compounds **3a** and **4** were characterized in a similar manner, and these spectral studies indicated that the nature of the fatty amine did not significantly influence the chemical shifts of the proton and carbon signals of the ATRA moiety.

Compound **4** was also characterized using spectral data. In the IR spectrum, characteristic bands at 3310 (N-H), 1638 (C=O) and 1227 cm^−1^ (C-N) were observed. The ^1^H-NMR showed resonances at 7.29 (s, 1H, NH), 6.72 (dd, 1H, C*^13^*H), 6.27 (m, 2H, C*^9^*H, C*^12^*H), 6.19 (d, 1H, C*^10^*H), 5.79 (s, 1H, C*^16^*H), 5.62 (s, 1H, C*^14^*H), 3.84–3.80 (m, 1H, CH of C_6_H_11_) and 3.63–3.61 ppm (m, 1H, CH of C_6_H_11_). These were further correlated with the signals observed in the ^13^C-NMR spectrum at 140.65, 137.24, 130.28, 127.48, 126.19, 125.70, 50.7 and 49.8 ppm.

### 2.2. Cytotoxicity

The cytotoxicity of compounds **3a**, **3b** and **4** was assessed using MTT and neutral red uptake (NRU) assays.

#### 2.2.1. MTT Assay

**Figure 1:**
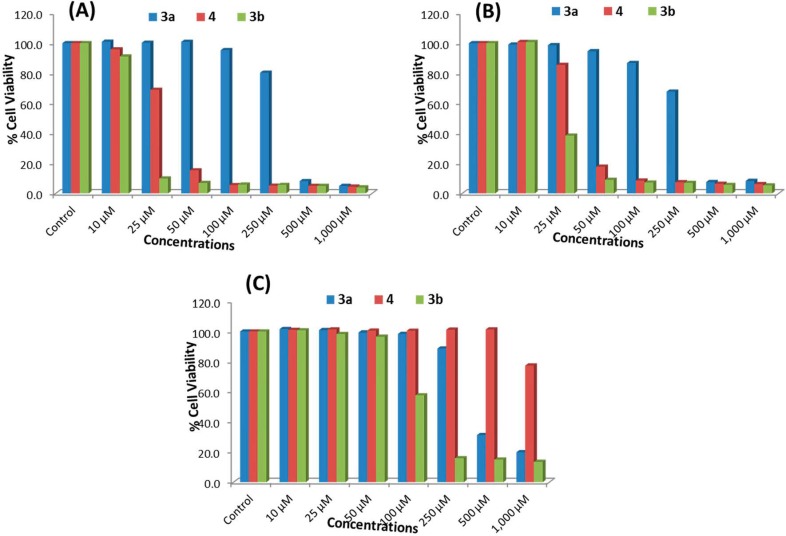
Cytotoxicity assessments by MTT assay in (**A**) MCF-7 cells, (**B**) HepG2 cells and (**C**) HEK293 cells. The cells were exposed to different concentrations of compounds **3a**, **3b** and **4** for 24 h. Values are the mean ± SE of three independent experiments.

The key results obtained using the MTT assay for ATRA and compounds **3a**, **3b** and **4** in MCF-7, HepG2 and HEK293 cells are shown in Table 1 and Figure 1A–C, respectively. A concentration-dependent decrease in cell viability was observed in MCF-7 and HepG2 cells following 24 h of exposure to compounds **3a**, **3b** and **4**. The cell viability at doses of 250, 500 and 1000 μM of compound **3a** was measured to be 80%, 8% and 5% in MCF-7 cells and 87%, 7% and 7% in HepG2 cells, respectively. Following treatment with 250-, 500- and 1000-μM doses of compound **4**, cell viability was measured to be 5%, 5% and 4% in MCF-7 cells and 7%, 6% and 6% in HepG2 cells, respectively. For compound **3b**, a decrease in the viability of MCF-7 and HepG2 cells was observed even after treatment with a dose of 25 μM Cell viability following doses of 25, 50, 100, 250, 500 and 1000 μM of compound **3b** was recorded as 10%, 7%, 6%, 6%, 5% and 5% in MCF-7 cells (Figure 1A) and 38%, 9%, 7%, 7%, 6% and 5% in HepG2 cells (Figure 1B), respectively. Compounds **3a** and **3b** were both less toxic to HEK293 cells than to MCF-7 and HepG2 cells. Compound **4** did not cause any cytotoxicity at lower doses in HEK293 cells, except at a dose of 1000 μM (Figure 1C). The viability of HEK293 cells following treatment with doses of 250, 500 and 1000 μM of compounds **3a**, **3b** and **4** was measured to be 88%, 31% and 19% (**3a**), 16%, 15% and 13% (**3b**), and 100%, 100% and 77% (**4**), respectively (Figure 1C). Compound **4** at 25, 50, 100, 250 and 500 M doses was found to induced cytotoxicity in cancer cells (MCF-7 and HepG2), but was found non-cytotoxic towards normal cells (HEK293). Similarly, compound **3b** showed cytotoxicity at a dose of 25 μM in MCF-7 and HepG2 cells, but not in HEK293 cells. The results also indicated that ATRA was not effective at concentrations ranging from 10–50 μM (Table 1).

**Table 1:** Cytotoxicity assessments of all trans-retinoic acid (ATRA) by MTT assay in MCF-7 cells, HepG2 cells and HEK293 cells. The cells were exposed to different concentrations of ATRA for 24 h. Values are the mean ± SE of three independent experiments.

Concentrations of ATRA	MCF-7 Cells	HepG2 Cells	HEK293 Cells
Control	100 ± 5.0	100 ± 1.6	100 ± 4.0
10 μM	104.0 ± 7.6	105.2 ± 1.7	102.6 ± 5.6
25 μM	99.0 ± 2.4	102.5 ± 4.3	102.2 ± 4.0
50 μM	97.0 ± 4.3	101.2 ± 3.2	100.2 ± 3.6
100 μM	93.9 ± 7.4	98.2 ± 3.4	99.1 ± 4.2
250 μM	90.5 ± 2.9	97.6 ± 2.2	97.2 ± 2.5
500 μM	86.8 ± 5.0	95.2 ± 6.0	98.4 ± 3.4
1000 μM	83.0 ± 5.8	91.9 ± 2.3	94.6 ± 4.9

#### 2.2.2. NRU Assay

**Figure 2:**
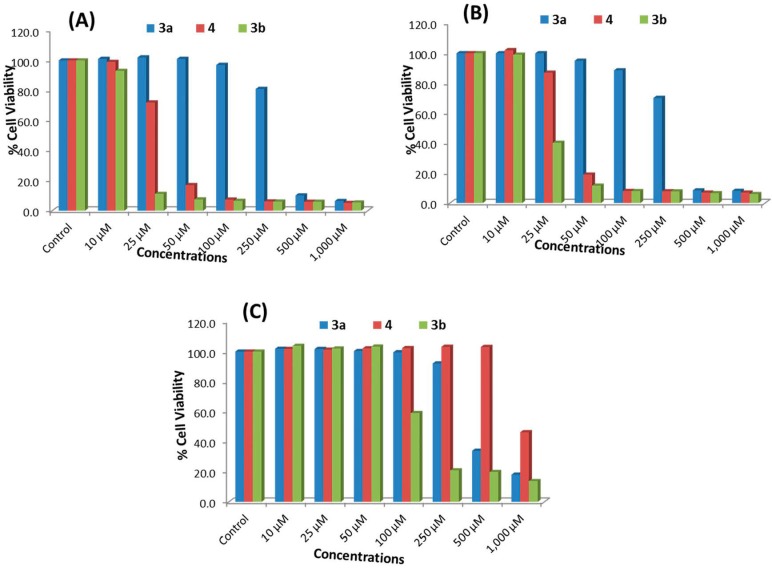
Cytotoxicity assessments by the neutral red uptake (NRU) assay in (**A**) MCF-7 cells, (**B**) HepG2 cells and (**C**) HEK293 cells. The cells were exposed to different concentrations of compounds **3a**, **3b** and **4** for 24 h. Values are the mean ± SE of three independent experiments.

The key results obtained using the NRU assay are summarized in Figure 2A–C. A dose-dependent decrease in the cell viability of MCF-7 and HepG2 cells was observed following treatment with compounds **3a**, **3b** and **4** for 24 h. The percentage of cell viability after 250-, 500- and 1000-μM doses of compound **3a** was measured to be 81%, 10% and 6% in MCF-7 cells and 70%, 9% and 8% in HepG2 cells, respectively. Following treatment with 250-, 500- and 1000-μM doses of compound **4**, the cell viability was found to be 6%, 6% and 5% in MCF-7 cells and 8%, 7% and 7% in HepG2 cells, respectively. For compound **3b**, a decrease in the cell viability of MCF-7 and HepG2 was recorded even after treatment with a 25-μM dose. The cell viability following treatment with 25-, 50-, 100-, 250-, 500- and 1000-Μm doses of compound **3b** was recorded as 11%, 8%, 7%, 6%, 6% and 5% in MCF-7 cells (Figure 2A) and 40%, 12%, 8%, 8%, 7% and 6% in HepG2 cells (Figure 2B), respectively. However, in HEK293 cells, the cell viability after treatment with 250-, 500- and 1000-μM doses of compounds **3a**, **3b** and **4** was found to be 92%, 34% and 18% (**3a**), 21%, 19% and 13% (**3b**) and 100%, 100% and 46% (**4**), respectively (Figure 2C). As in the MTT assay, compound **3a** was found to be less cytotoxic than **3b**, and compound **4** did not cause any cytotoxic affect except at a 1000-μM concentration, as compared to the untreated control (Figure 2C). Compound **4** at 25-, 50-, 100-, 250- and 500-μM doses was also found to induce cytotoxicity in cancer cells (MCF-7 and HepG2) by the NRU assay, but was found non-cytotoxic towards normal cells (HEK293). Compound **3b** also was found to induce cytotoxicity at a dose of 25 μM in MCF-7 and HepG2 cells, but not in normal HEK293 cells by the NRU assay.

The results of the MTT and NRU assays showed that compound **3b** exhibited the highest cytotoxicity against both types of cancer cells, and compound **4** was the second most effective. Compound **3a** was found to be the least effective, and significant cytotoxicity was observed only at higher concentrations, *i.e.*, 250 μM or above. The MTT assay was a more effective representative of cytotoxicity than the NRU assay. It has previously been reported that the results can vary depending on the cytotoxicity assay used [26].

### 2.3. Morphological Analysis in MCF-7, HepG2 and HEK293 Cells

**Figure 3:**
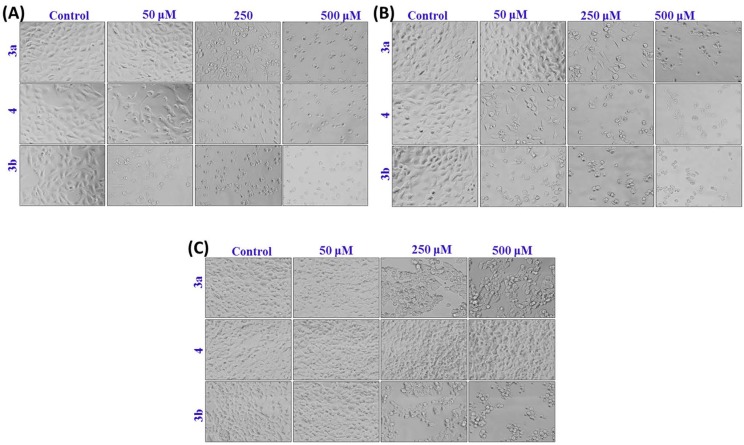
Morphological changes in (**A**) MCF-7 cells, (**B**) HepG2 cells and (**C**) HEK293 cells. The cells were exposed to different concentrations of compounds **3a**, **3b** and **4** for 24 h. Images were taken using an inverted phase contrast microscope at 20× magnification.

The morphological changes observed in cells that were exposed to compounds **3a**, **3b**, and **4** for 24 h are shown in Figure 3A–C. Changes in morphology were observed using a phase contrast inverted microscope. The cells indicate that the most prominent effects after the exposure of compounds and changes in their morphology were found to be concentration dependent. Cells exposed to higher doses of compounds lose their normal morphology and shape; these cells become more rounded and less adherent than the control.

### 2.4. Flow Cytometry Analysis

**Figure 4:**
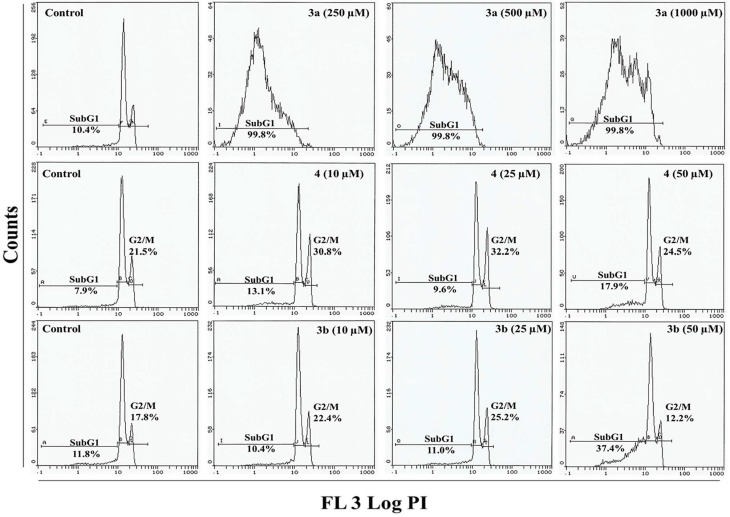
Flow cytometry images of treated HepG2 cells. Flow cytometry images show changes in cell cycle progression in HepG2 cells exposed to compounds **3a**, **3b** and **4** for 24 h. Sub-G1 indicates the cells undergoing apoptosis/necrosis and G2/M indicates the cell cycle arrest.

**Figure 5:**
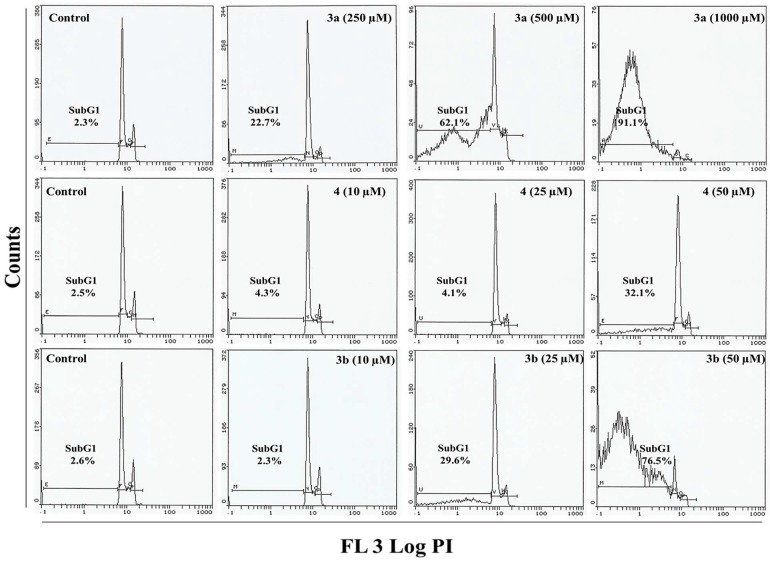
Flow cytometry images of treated MCF-7 cells. Flow cytometry images depicting changes in cell cycle progression in MCF-7 cells exposed to compounds **3a**, **3b** and **4** for 24 h. Sub-G1 indicates the cells undergoing apoptosis/necrosis.

In order to evaluate the anticancer properties of compounds **3a**, **3b** and **4**, we studied the cell death of HepG2 and MCF-7 cells. Cell cycle analysis of cells treated with compounds **3a**, **3b** and **4** indicated an increase in the apoptotic subG_1_ population after 24 h of exposure (Figure 4 and Figure 5). With a 10.4% background level of apoptosis in the HepG2 control, compound **3a** at its lowest treatment concentration (250 µM) clearly induced a great deal of cell death, as evidenced by the appearance of 99.8% dead cells in the sub-G_1_ phase (Supplementary Figure S1). HepG2 cells exposed to low doses of compound **4** exhibited a pattern typical of G_2_/M arrest. In control cells, 21.5% of control cells were in the G_2_/M phase; after treatment with 10- and 25-µM doses of compound **4**, 30.8% and 32.2% cells were in the G_2_/M phase. At the highest concentration (50 µM), compound **4** led to the transition of G_2_/M-arrested cells (24.5%) to the apoptotic phase sub-G1 (17.9%). A similar pattern of G_2_/M arrest was observed in HepG2 cells treated with compound **3b**. Following treatment with low doses (10 and 25 µM) of **3b**, 22.4% and 25.2% of cells were in the G_2_/M phase compared to 17.8% in the untreated control. Compound **3b** at the highest concentration (50 µM) induced a stronger apoptotic response with 37.4% cells in the sub-G_1_ phase (Supplementary Figure S1). Compared to HepG2, a different change in cell cycle progression was observed in MCF-7 cells (Figure 5). Cells exposed to 250- and 500-µM doses of compound **3a** had 22.7% and 62.1% cells in the sub-G_1_ phase, while 91.1% cells were in the sub-G_1_ apoptotic phase following treatment with a 1000-µM dose. Compounds **3b** and **4** at 50 µM significantly induced apoptosis in MCF 7 cells as evidenced by the 76.5% and 32.1% of cells in the sub-G1 phase, respectively (Figure S2). However, low concentrations of compound **3b** or **4** did not induce G_2_/M arrest in MCF-7.

Analysis of the effect of compound **3a** on HepG2 and MCF-7 cells suggests that compound **3a** preferentially induces apoptosis in HepG2 cells at a concentration of 250 µM. On the other hand, we observed more cell death in HepG2 and MCF-7 cells with low concentrations of **3b** and **4**. Compounds **3b** and **4** killed HepG2 cells predominantly by arresting them in the G_2_/M phase, which is followed by apoptosis. Compounds **4** and **3b** kill significant numbers of MCF-7 cells by inducing apoptotic/necrotic events. The appearance of a sub-G_1_ peak in the cell cycle analysis after the concentration of compounds **4** and **3b** was increased suggests the upregulation of the early and late apoptotic/necrotic pathway, which might be induced by a change in mitochondrial and lysosomal function [27,28]. Additionally, HepG2 cells underwent G_2_/M arrest following treatment with compound **4**, reflecting the strong possibility of heavy DNA damage and failure of the DNA repair machinery in cells. It is known that the cellular DNA repair mechanisms are highly conserved [29] and that extensive DNA damage may lead to cell-cycle arrest and cell death [30,31].

### 2.5. Apoptosis/Necrosis Assessment Using Annexin V-PE and 7-AAD in HepG2 and MCF 7 Cells

**Figure 6:**
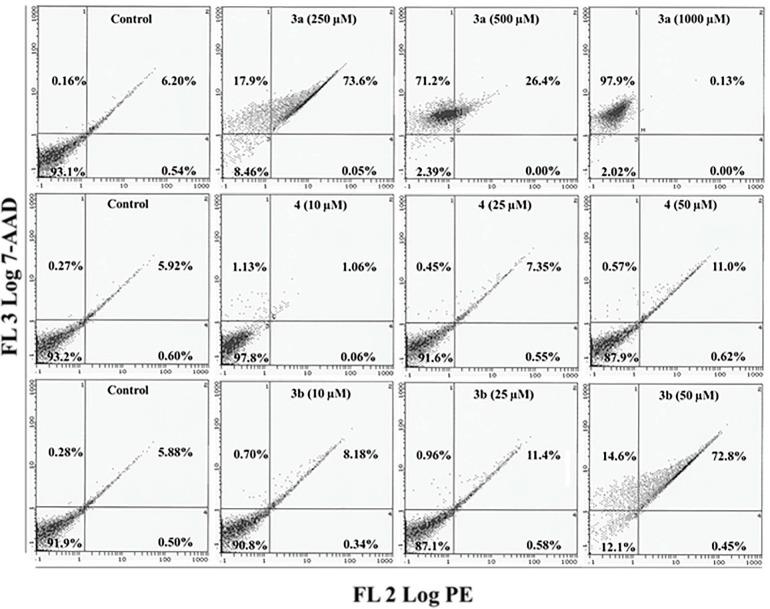
Annexin V-PE (phycoerythrin) and 7-AAD (7-aminoactinomycin D) assay following treatment of HepG2 cells. Bivariate flow cytometry analysis of HepG2 cells treated with compounds **3a**, **3b** and **4**. Early apoptotic, late apoptotic and necrotic cells following 24 h treatment are shown by the scatter plots.

The flow cytometry data with Annexin V-PE (phycoerythrin) and 7-aminoactinomycin D (7-AAD) clearly demonstrated that compounds **3a**, **3b** and **4** predominantly trigger cell death by initiating early and late apoptotic processes with simultaneous transformation into the necrotic phase. Cell death induced by compounds **3a**, **3b** and **4** was further confirmed using the Annexin V-PE and 7-AAD apoptotic assays. The results are represented in a scatter plot, which represents the fluorescence generated by Annexin V-PE interacting with phosphatidylserine on the plasma membrane of apoptotic/necrotic cells and the specific staining of necrotic cells with 7-AAD dye. Based on the Annexin V-PE and 7-ADD staining, more than 91.9% of HepG2 control cells were found alive with 0.5%, 5.8% and 0.2% of cells classified as being in the early, late and necrotic stages. Exposure of HepG2 cells to 250-, 500- and 1000-µM doses of compound **3a** resulted in the induction of necrosis, which is represented by a shift of 17.9%, 71.2% and 97.9% of the cellular population into the upper left quadrant (Annexin V−/7-AAD+) of the plot, which represents necrotic cells (Figure 6). HepG2 cells that were exposed to low concentrations of 25 and 50 µM of compound **4** induced late apoptosis with an increase of 7.35% and 11% of cells in the upper right quadrant (Annexin V+/7-AAD+), which represents late apoptotic cells. In contrast, the exposure of HepG2 cells to 10-, 25- and 50-µM doses of compound **3b** induced a concentration-dependent increase of 8.1%, 11.4% and 72.8% of cells in the late apoptotic quadrant. In addition, the highest concentration (50 µM) of compound **3b** shifted 14.6% of cells into the upper left necrotic quadrant (Figure 6).

**Figure 7:**
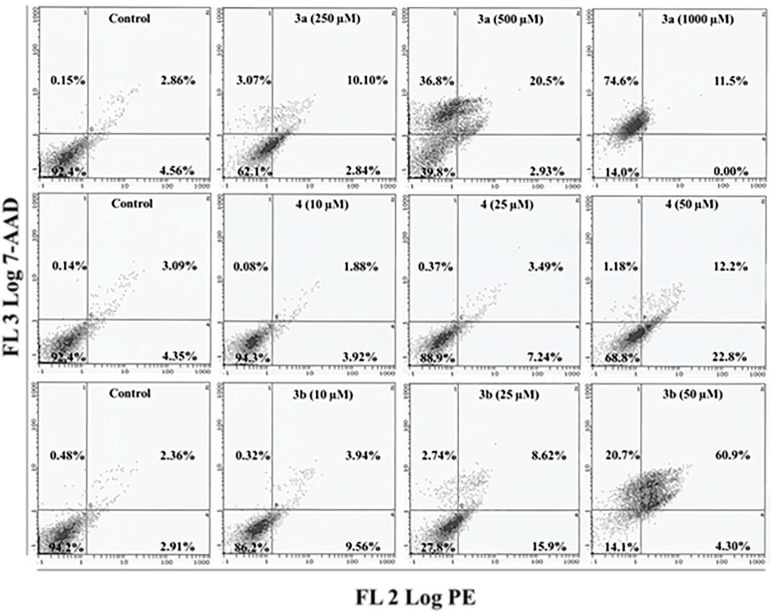
Annexin V-PE (phycoerythrin) and 7-AAD (7-aminoactinomycin D) assay following treatment of MCF-7 cells. Bivariate flow cytometry analysis of MCF-7 cells treated with compounds **3a**, **3b** and **4**. Early apoptotic, late apoptotic and necrotic cells following 24 h treatment are shown by the scatter plots.

Compared to HepG2, the Annexin-V results of MCF-7 cells further validated its unique response towards compounds **3a**, **3b** and **4**. MCF-7 cells exposed to doses of 250, 500 and 1000 µM of compound **3a** exhibited only 3.0%, 36.8% and 74.6% of cells in the quadrant representing necrotic cells (Figure 7). Exposure to compound **4** resulted in the shift of MCF-7 cells towards the lower right quadrant (Annexin V+/7-AAD−), indicating the onset of the early apoptotic phase. After doses of 10, 25 and 50 µM of compound **4**, 3.9%, 7.2% and 22.8% of cells were observed in the lower right quadrant (Figure 7). Likewise, MCF-7 cells that were exposed to low doses of compound **3b** (10 and 25 µM) also induced early apoptotic events as evidenced by the appearance of 9.5% and 15.9% of cells in the lower right quadrant. At the highest concentration (50 µM) of **3b**, 60.9% and 20.7% of MCF-7 cells gradually shifted to the late apoptotic quadrant and the necrotic quadrant (Figure 7). Compared to compound **4**, compound **3b** significantly increased the number of early apoptosis events observed in the Annexin V+/7-AAD− quadrant, which signifies the disintegration of the plasma membrane and externalization of phosphatidylserine (PS), an event indicative of early apoptosis [32,33,34]. However, the appearance of a significant population of late apoptotic cells suggests the complete loss of the cell membrane integrity, eventually causing cell death due to necrosis in compound **3b**-exposed cancerous cells. Overall, our flow cytometry data suggests that compound **3b** possesses a greater anticancer activity in MCF-7 cells than in HepG2 cells.

## 3. Experimental Section

### 3.1. Reagents

ATRA, undecylamine, octadecylamine, oleylamine, *N,N'*-dicyclohexylcarbodiimide (DCC), 4-dimethylaminopyridine (DMAP) and all other chemicals were purchased from Sigma, St. Louis, MO, USA. For thin-layer chromatography (TLC), Silica Gel F254 plates, Merck, NJ, USA (20 × 5cm) were used. The plates were eluted using a mixture of petroleum ether, CHCl_3_ and CH_3_COOH (70:30:1). Silica gel 230–400 mesh (Merck, NJ, USA) was used for column chromatography. Dulbecco’s Modified Eagle’s Medium (DMEM), antibiotic/antimycotic solutions and fetal bovine serum (FBS) were obtained from Invitrogen, Life Technologies, Waltham, MA, USA. A Shimadzu 8201 PC spectrophotometer, a Bruker DRX 400 spectrometer and a JEOL-SX 102/DA-600 mass spectrometer were used to obtain spectral data.

### 3.2. Synthesis of Amides (**3a–b**) and 1-Substituted-1,3-dicyclohexylurea (**4**) Derivatives of ATRA

The synthesis of the target compounds was achieved by dissolving a long chain fatty amine (0.011 mol), ATRA (0.011 mol) and DCC (0.011 mol) in DMSO (50 mL). A catalytic amount of DMAP (0.001 mol) was added, and the solution was stirred at room temperature. After completion of the reaction, the reaction mixture was filtered (to remove solid dicyclohexylurea), and the filtrate was dried under reduced pressure. The amidic derivatives **3a** and **3b** and the acyl urea derivative **4** of ATRA were obtained by column chromatography using gradient elution with hexane and chloroform. The identity of the desired compounds was confirmed using infrared (IR) and ^1^H and ^13^C nuclear magnetic resonance (NMR) spectroscopy and mass spectrometry as shown below.

*N-undecyl-retinamide* (**3a**): Yield = 45%; IR (cm^−1^): 3304 (N-H), 2921 (C-H asymmetric), 2847 (C-H symmetric), 1630 (C=O); ^1^H-NMR (CDCl_3_, δH): 7.27 (1H, s, NH), 6.74 (1H,dd, C*^13^*H), 6.21 (2H, m, C*^9^*H, C*^12^*H), 6.17 (1H, d, C*^10^*H), 5.72 (1H,s, C*^16^*H), 5.62 (1H, s, C*^14^*H), 2.18 (2H, m, C*^6^*H_2_), 2.16 (3H, s, C*^21^*H_3_), 1.91 (3H, s, C*^20^*H_3_), 1.64 (3H, s, C*^19^*H_3_), 1.56 (2H, s, O=C-NH-CH_2_), 1.51(2H, m, C*^1^*H_2_), 1.49 (2H, m, C*^2^*H_2_), 1.46 (2H,m, O=C-NH-CH_2_-CH_2_), 1.29 (16H, br.s., chain CH_2_), 0.83 (3H, distorted t, terminal CH_3_);^13^C-NMR (CDCl_3_, δC):163.6, 152.3, 140.6, 137.7, 137.4, 137.2, 130.3, 130.2, 127.5, 126.1, 125.7, 40.5, 39.30, 33.83, 32.67, 31.82, 29.71, 29.62, 29.55, 29.56, 29.51, 29.47, 29.42, 29.28, 27.07, 22.59, 21.28, 19.00, 18.55, 14.09, 13.99; ESI-MS: [M + Na]^+^ experimental = 476.74, C_31_H_51_NO calculated = 476.73. Anal. calcd. for C_31_H_51_NO: C, 82.06; H, 11.31; N, 3.08. Found: C, 82.09; H, 11.35; N, 3.06.

*N-oleyl-retinamide* (**3b**): Yield = 44%; IR (cm^−1^): 3302 (N-H), 2927 (C-H asymm.), 2848 (C-H symm.), 1633 (C=O); ^1^H-NMR (CDCl_3_, δH): 7.25 (1H, s, NH), 6.79 (1H, dd, C*^13^*H), 6.23 (2H, m, C*^9^*H, C*^12^*H), 6.09 (1H, d, C*^10^*H), 5.74 (1H, s, C*^16^*H), 5.65 (1H, s, C*^14^*H), 5.30 (2H, m, CH=CH), 2.18 (2H, m, C*^6^*H_2_), 2.21 (3H, s, C*^21^*H_3_), 1.96 (4H, m, CH_2_-CH=CH-CH_2_), 1.93 (3H, s, C*^20^*H_3_), 1.67 (3H, s, C*^19^*H_3_), 1.50 (2H, m, O=C-NH-CH_2_), 1.58 (2H, m, C*^1^*H_2_), 1.48 (2H, m, C*^2^*H_2_), 1.48 (2H, m, O=C-NH-CH_2_-CH_2_), 1.23 (12H, br.s., 6CH_2_), 1.12 (10H, br.s., chain 5CH_2_), 0.84 (3H, dist. t, terminal CH_3_); ^13^C-NMR (CDCl_3_, δC):166.8, 152.3, 142.8, 140.6, 137.7, 137.2, 130.8, 130.3, 130.0, 130.0, 129.5, 126.1, 125.7, 39.53, 39.30, 36.13, 32.45, 31.84, 29.70, 29.65, 29.46, 29.42, 29.31, 29.26, 29.24, 29.23, 29.11, 29.05, 28.62, 28.61, 27.77, 27.14, 22.62, 20.96, 19.00, 18.94, 14.04, 12.12; ESI-MS: [M + Na]^+^ experimental = 572.92, C_38_H_63_NO calculated = 572.90. Anal. calcd. for C_38_H_63_NO: C, 83.00; H, 11.57; N, 2.54. Found: C, 83.04; H, 11.56; N, 2.58.

*1-(Retinoyl)-1,3-dicyclohexylurea* (**4**): Yield = 46%; IR (cm^−1^): 3310 (N-H), 2929 (C-H asymm.), 2852 (C-H symm.), 1638 (C=O), 1227 (C-N); ^1^H NMR (CDCl_3_, δH): 7.29 (1H, s, NH), 6.72 (1H, dd, C*^13^*H), 6.27 (2H, m, C*^9^*H, C*^12^*H), 6.19 (1H, d, C*^10^*H), 5.79 (1H, s, C*^16^*H), 5.62 (1H, s, C*^14^*H), 3.84–3.80 (1H, m, CH of C_6_H_11_), 3.63–3.61 (1H, m, CH of C_6_H_11_, 2.23 (2H, m, C^6^H_2_), 2.16 (3H, s, C*^21^*H_3_), 1.94 (3H, s, C^20^H_3_), 1.67 (3H, s, C^19^H_3_), 1.54 (2H, m, C^1^H_2_), 1.45 (2H, m, C^2^H_2_), 1.37–1.35 (20H, m, 10CH_2_); ^13^C-NMR (CDCl_3_, δC): 165.8, 159.3, 152.3, 140.6, 137.7, 137.2, 130.2, 130.2, 127.4, 126.1, 125.7, 50.7, 49.8, 39.27, 33.84, 33.37, 33.32, 32.66, 30.90, 30.74, 28.65, 28.64, 26.29, 25.76, 25.52, 25.48, 24.95, 24.85, 21.29, 18.97, 18.40, 12.41; ESI-MS: [M + Na]^+^ experimental = 506.77, C_33_H_50_N_2_O_2_ calculated = 506.76. Anal. calcd. for C_33_H_50_N_2_O_2_: C, 78.22; H, 9.93; N, 5.52. Found: C, 78.26; H, 9.91; N, 5.53.

### 3.3. Cell Culture

MCF-7 (breast cancer), HepG2 (liver cancer) and HEK293 (embryonic kidney) cells were obtained from American Type Culture Collection (ATCC; Manassas, VA, USA). Cells were grown in DMEM with 10% FBS in 5% CO_2_ at 37 °C under high humidity. The trypan blue dye exclusion test [35] was used to assess cell viability. Only cells that showed a viability of more than 98% were used in this study.

### 3.4. 3-(4,5-Dimethylthiazol-2-yl)-2,5-diphenyltetrazolium Bromide Assay

Cell viability was assessed using the MTT assay [35]. In brief, 1 × 10^4^ cells were plated in 96-well plates and allowed to adhere to the wells overnight in a CO_2_ incubator. After treatment, MTT (10 µL) was added to each well and incubated in a CO_2_ incubator for 4 h. Then, the supernatant was discarded, and DMSO (200 μL) was added to each well. The absorbance was read at a wavelength of 550 nm.

### 3.5. Neutral Red Uptake Assay

An NRU assay was performed according to the protocol described by Siddiqui *et al.* [36]. Post treatment, the cells were washed with phosphate-buffered saline (PBS; 0.01 M; pH 7.4), and 50 µg/mL of neutral red containing medium were added. The cells were then subjected to 3 h of incubation. The supernatant was removed, and the cells were washed with a solution of 0.5% CH_2_O and 1% CaCl_2_. Subsequently, a solution of 1% CH_3_COOH and 50% EtOH was added, and the dye was extracted. The absorbance was then read at a wavelength of 550 nm.

### 3.6. Morphological Analysis Using Phase Contrast Microscopy

Changes in morphology were observed to determine the effect of the novel compounds in MCF-7, HepG2 and HEK293 cells. The cells were exposed to different concentrations (10–1000 μM) of compounds **3a**, **3b** and **4** for 24 h. The images were recorded using an inverted phase contrast microscope at 20× magnification.

### 3.7. Cell Cycle Analysis

Measurement of cell cycle arrest was performed using the method of Saquib *et al.* [37]. Briefly, HepG2 and MCF-7 cells were exposed to various concentrations of compounds **3a**, **3b** and **4** for 24 h. After centrifugation for 4 min at 1000 rpm, cells were fixed with 70% ethanol (500 µL) and were then incubated at 4 °C for 1 h. The cells were then washed and stained using PBS (500 µL; 0.01 M; pH 7.4) containing 50 µg/mL propidium iodide (PI), 0.5 mg/mL RNase and Triton X-100. The PI fluorescence was measured using a Beckman Coulter flow cytometer. The results were analyzed using Coulter Epics XL/XL-MCL, System II Software (Version 3.0, Beckman Coulter, Inc. 250 S. Kraemer Blvd. Brea, CA).

### 3.8. Apoptosis/Necrosis Assay Using Annexin V-PE and 7-Aminoactinomycin D

The assay was performed according to the manufacturer’s instructions using Annexin V-PE and 7-AAD (Beckman Coulter, Marseille, Cedex 9, France) kits. Briefly, MCF-7 and HepG2 cells were exposed to 250, 500 and 1000 µM of compound **3a** and 10, 25 or 50 µM of **3b** and **4** for 24 h. The amount of apoptosis/necrosis in the treated HepG2 and MCF-7 cells was assessed by flow cytometry using the detailed reported protocol [38].

### 3.9. Statistical Analysis

ANOVA was used for statistical analysis, and results are expressed as the mean ± standard error of three separate experiments. The treated and control groups were compared using the *post hoc* Dunnett’s test. A value of *p* < 0.05 was considered statistically significant.

## 4. Conclusions

This article describes the synthesis and characterization of novel amidic and acyl urea derivatives of ATRA. The cytotoxicity measurements demonstrated a concentration-dependent reduction in the cell viability and alteration of cellular morphology in MCF-7 and HepG2 cells, whereas, in our hands, ATRA was not effective at concentrations ranging from 10–50 µM. The use of flow cytometry to assess cell cycle progression and an apoptosis assay using Annexin V-PE and 7-AAD also revealed that the novel ATRA derivatives possess substantial anticancer activity towards human cancer cell lines (MCF-7 and HepG2). Arrest in the G_2_/M phase of the cell cycle could be one of the mechanisms by which cell growth is inhibited and apoptosis is induced by the compounds.

## Supplementary Materials

Supplementary can be accessed at: http://www.mdpi.com/1420-3049/20/05/8181/s1.
