# Supplementary material for: Novel All Trans-Retinoic Acid Derivatives: Cytotoxicity, Inhibition of Cell Cycle Progression and Induction of Apoptosis in Human Cancer Cell Lines
Source: Molecules. 2015 May 7;20(5):8181–97. doi: 10.3390/molecules20058181 (PMC6272518; doi:10.3390/molecules20058181)
Supplement: Supplementary file 1 [file molecules-20-08181-s001.pdf]

Supplementary Materials

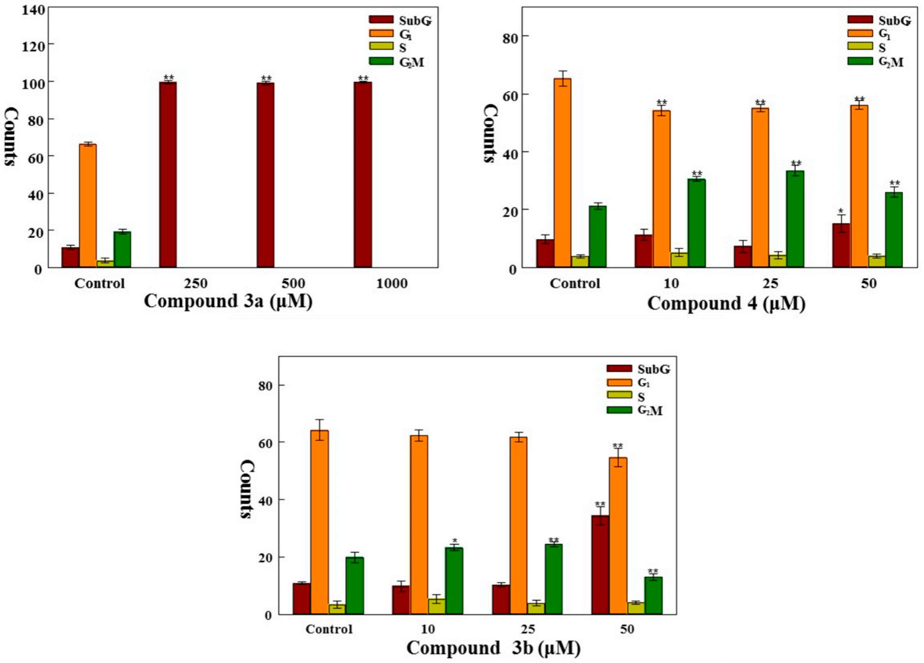

Figure S1. Histogram showing flow cytometry data of treated $HepG_2$ cells. The percentage of cells in each phase of the cell cycle is represented by G_1, S, G_2/M, and the percentage of cells that have undergone apoptosis is represented by $subG_1$. Each histogram represents data obtained from three independent experiments. Mean ± SD of different phases of cell cycle arrest in $HepG_2$ cells. * $p < 0.05$, ** $p < 0.05$ versus control.

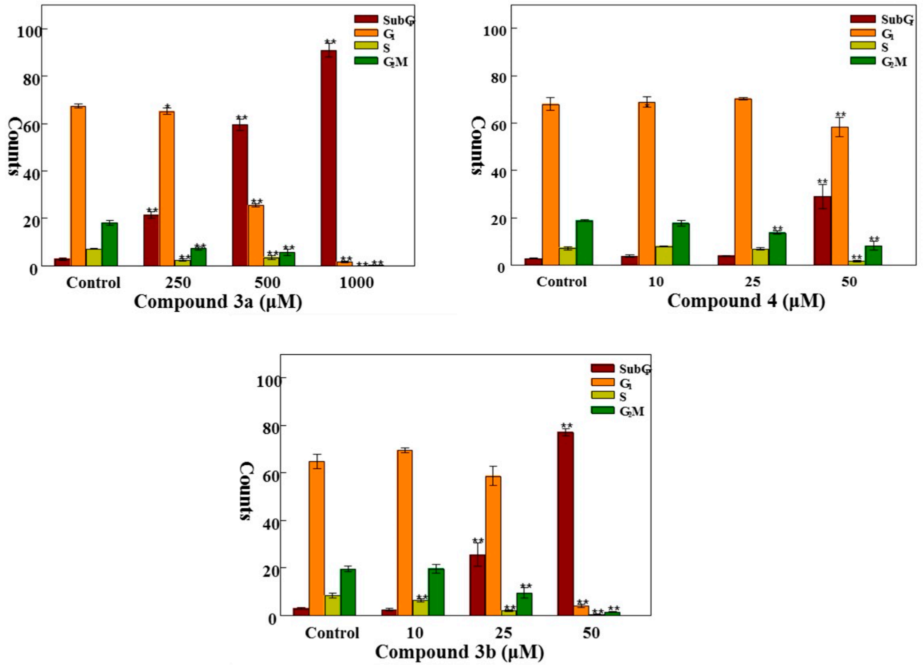

Figure S2. Histogram showing flow cytometry data of treated MCF-7 cells. The percentage of cells in each phase of the cell cycle is represented by G_1, S, G_2/M, and the percentage of cells that have undergone apoptosis is represented by $subG_1$. Each histogram represents data obtained from three independent experiments. Mean ± SD of different phases of cell cycle arrest in $HepG_2$ cells. * $p < 0.05$, ** $p < 0.05$ versus control.
